# An eQTL biological data visualization challenge and approaches from the visualization community

**DOI:** 10.1186/1471-2105-13-S8-S8

**Published:** 2012-05-18

**Authors:** Christopher W Bartlett, Soo Yeon Cheong, Liping Hou, Jesse Paquette, Pek Yee Lum, Günter Jäger, Florian Battke, Corinna Vehlow, Julian Heinrich, Kay Nieselt, Ryo Sakai, Jan Aerts, William C Ray

**Affiliations:** 1 The Research Institute at Nationwide Children's Hospital, Columbus OH, USA; 2 Ayasdi Inc. Palo Alto CA, USA; 3 Center for Bioinformatics Tübingen, University of Tübingen, Germany; 4 VISUS, University of Stuttgart, Stuttgart, Germany; 5 ESAT-SCD/IBBT-KU Leuven Future Health Department, Leuven University, Leuven, Belgium; 6 The Ohio State University Biophysics Program, Columbus OH, USA

## Abstract

In 2011, the IEEE VisWeek conferences inaugurated a symposium on Biological Data Visualization. Like other domain-oriented Vis symposia, this symposium's purpose was to explore the unique characteristics and requirements of visualization within the domain, and to enhance both the Visualization and Bio/Life-Sciences communities by pushing Biological data sets and domain understanding into the Visualization community, and well-informed Visualization solutions back to the Biological community. Amongst several other activities, the BioVis symposium created a data analysis and visualization contest. Unlike many contests in other venues, where the purpose is primarily to allow entrants to demonstrate tour-de-force programming skills on sample problems with known solutions, the BioVis contest was intended to whet the participants' appetites for a tremendously challenging biological domain, and simultaneously produce viable tools for a biological grand challenge domain with no extant solutions. For this purpose expression Quantitative Trait Locus (eQTL) data analysis was selected. In the BioVis 2011 contest, we provided contestants with a synthetic eQTL data set containing real biological variation, as well as a spiked-in gene expression interaction network influenced by single nucleotide polymorphism (SNP) DNA variation and a hypothetical disease model. Contestants were asked to elucidate the pattern of SNPs and interactions that predicted an individual's disease state. 9 teams competed in the contest using a mixture of methods, some analytical and others through visual exploratory methods. Independent panels of visualization and biological experts judged entries. Awards were given for each panel's favorite entry, and an overall best entry agreed upon by both panels. Three special mention awards were given for particularly innovative and useful aspects of those entries. And further recognition was given to entries that correctly answered a bonus question about how a proposed "gene therapy" change to a SNP might change an individual's disease status, which served as a calibration for each approaches' applicability to a typical domain question. In the future, BioVis will continue the data analysis and visualization contest, maintaining the philosophy of providing new challenging questions in open-ended and dramatically underserved Bio/Life Sciences domains.

## Introduction

The biological sciences have a uniquely intertwined yet strangely dysfunctional relationship with the bioinformatics and visualization sciences. Bio/Life Sciences researchers and practitioners regularly rely on visualization techniques for solving a large range of problems, including use of charts, graphs and interactive displays. They frequently prefer these visualization techniques to analytical techniques, methods of a computational and/or statistical nature, even when the analytical techniques produce more accurate results. For example every biochemistry student knows how to calculate rate constants for Michaelis-Menten [1] enzyme kinetics based on extracting the slope and intercept from a hand fitted double reciprocal Lineweaver-Burk plot [2]. Despite years of understanding that the double reciprocal plot distorts errors, making accurate hand fitting of the data almost impossible [3], this and other problematic graphical linearizations are still in use. At the same time, most students would be hard-pressed to write down the appropriate regression framework to calculate these constants analytically. The extreme inertia of such visual representation and problem solving methods in the biological sciences is not solely limited to approaches developed before the advent of modern high-speed computers. Despite a direct statement that his clustering and visualization methods were simply a first attempt at analyzing MicroArray data, the hierarchical clustering and heat map visualization from Michael Eisen's seminal 1998 paper on microarray clustering [4], remain a de facto standard which is only slowly being questioned today [5].

Additional and profound examples of this odd relationship can be seen between bioinformatics and biology as well. However enticing the size and rich complexity of data sets produced by their biological peers, computational experts may be less excited by the prospect of acquiring and encoding all of the domain knowledge necessary to develop tools that are optimized to a biological need. As a result, biological researchers often conclude that many computational contributions to biological data analysis are driven more by what is computationally interesting, or computationally expedient, than by what is biologically appropriate. For example, a seminal and ubiquitous computational strategy for identifying sequence similarity, the BLAST algorithm, ranks search results based not on the likelihood of biological relationship, which is how the typical working biologist applies the results, but on a p-value-like statistic that ranks results approximately based on the reciprocal of the probability that the relationship occurred randomly [6]. The ubiquitous p-value itself, long understood to be a biased measure of effect size and not a measure of the strength of the evidence provided by a given dataset, despite the fact that those are the most common uses of the p-value, is only recently coming under fire as a problematic player that needs to be fixed, rather than a suboptimal solution that needs to be lived with [7].

In this environment of interdependence across three disciplines with frequently misaligned goals, there is the opportunity for a constant undercurrent of miscommunication. When computationalists are asked to provide visualization tools for molecular motion using a representation that's visually orthogonal to everything known about representing complex motion in other motion-intense fields such as Computational Fluid Dynamics(CFD) [8], and biologists, unaware of the lessons from CFD, repeatedly request tools using this paradigm, both groups quite rightly recognize that something has gone wrong, but neither has the perspective to identify the problem. Other examples abound, and quite frequently the result of collaborations on the part of the bio/life sciences and computational sciences, is an unused tool and hurt feelings all around. Yet even as problematic as the intersection of these fields is, their marriage is also one of the great opportunities facing the community of Visualization, Bioinformatic, and Bio/Life Sciences in the future. Rapid advances in raw computing power and graphics processing power make visualization approaches that could only be dreamed about a few years ago, available on commodity desktop platforms. At the same time, next-generation sequencing, and other biological data-acquisition technologies are creating new data types and data sets that researchers cannot hope to approach without revolutionary advances in visualization and summarization. It is into this world of past failures, near misses, occasional brilliant successes, and an exponentially growing, overwhelming oncoming need, that the BioVis symposium was born. It is clear that contributions to the domain will need to be shepherded carefully; An inappropriate representation that is adopted by the biological domain because it stands alone in the field at the outset, could, if history is an indicator, leave biologists using a crippled tool for decades; One poorly explained biological need, and a flotilla of inappropriate representations could be launched into use. The Visualization expert holds a unique position in this world, where their principled approach to appropriate information representation, and quantitative concern for accurate information transfer, can be applied to the benefit of all three fields. In recognition of the opportunity, and responsibility of that position the BioVis data analysis and visualization contest was designed.

Our goals for the contest were threefold:

1. The development of a better-informed Vis community, provided with deeper domain-specific intuition into the actual issues of interest to the user community.

2. A better-tooled biological community, provided with enhanced applications specifically adjusted to meet their analysis needs.

3. Finally, a mechanism to strongly promote fundable peer collaborations between Visualization and Bio/Life-Sciences researchers.

To meet these goals, the contest was designed so that each theme spanned multiple symposia years, with an outgoing advanced phase, and an incoming basic phase running in parallel. In 2011, our first year, we ran only the basic challenge. During the contest, a web forum was available for discussion of the challenge domain in general as well as specifics of the challenge data, both between teams and with contest-provided domain experts. At the symposium, a session was available for entrants to discuss their entries with each other and with the contest judges and the domain expert (Chris Bartlett) who created the data. Future advanced-phase challenges will provide entrants the opportunity to further develop their basic-phase approaches based on feedback and new insights gained during the contest and via the symposium session.

## Biological domain

A new biological domain will be introduced each year and retained for two years. The first year is a Phase I practice dataset to introduce the topic while the second year, Phase II, dataset is larger in scope and size. Every year after 2011, there will be two concurrent contests, one for Phase I and one for Phase II. As 2011 is the inaugural year, the only contest was Phase I of expression Quantitative Trait Locus (eQTL) data [9]. eQTL experiments catalog massive collections of correlated genotype and phenotype data, in the hope of detecting important genome-sequence variations that affect RNA expression levels and identifying the underlying mechanisms. The mechanisms often are networks of interacting polymorphisms that non-linearly affect specific expression levels, conditional on the presence of (possibly multiple) other polymorphisms, and on the tissue type in which they are acting. eQTL analysis is a nascent field, because broad-coverage genotype and expression surveys are only just becoming feasible. It is an attractive field for Visualization and Visual Analytics, both because it is a "hot topic," and because it is a phenomenally data-rich and information-dense domain. A typical complete analysis will eventually survey a few million genotypic loci and tens of thousands of expression levels, differentially in up to 100 tissue types, across 1000 or more subjects [10]. The magnitude and complexity of this data absolutely demands sophisticated mechanisms for summarizing and presenting the data. eQTL analysis is also an ideal model for the emerging field of personalized medicine, because the decision support problem for Personalized Medicine "have I considered all of the important factors in making this decision?" is exactly the same as the eQTL interaction-network-discovery problem of "have I identified all of the relevant interacting factors?". Our contest data was generated from actual published and publicly available eQTL data, using an observation-shuffling technique. This technique preserved the biological complexity of the data, while allowing us to "spike in" a network of synthetic interactions for the purpose of establishing specific items of ground truth for contestants to find.

Because our goals are to encourage and enable the Visualization community to produce tools that are highly relevant to the Bio/Life-Sciences community, it was important that we maintain realistic complexity within the data. By maintaining realism, we assure that tools that address the contest data, are directly relevant for real data, and we enhance our participants' appreciation of the depth and breadth of opportunity in the domain. Simultaneously, because the tools produced are immediately useful and relevant, our approach encourages the Bio/Life-Sciences community to better-engage the Visualization community.

### Visualization and analytical complexity

eQTL analysis provides a target-rich domain for visualization and visual analytics approaches. With the goal of "convey how it works", across data with potentially millions of variables, just the sheer size makes visual abstraction and summarization a practical necessity. The complex and conditional interrelations, and the necessity of communicating these as a goal, further cements the importance of visualization to this domain. While one might think of an eQTL data set as being represented by a graph with nodes representing genomic loci, and edges representing relationships, the requirements for eQTL analysis and representation go beyond traditional network/graph representation techniques, and no extant technique is completely adequate to convey the conditional, and biologically error-laden results.

Even raw statistical analysis of this data is problematic. It is fairly easy to analyze single-locus direct effects where, all other things being equal, the presence of a particular allele at some locus predisposes an expression level to be elevated or depressed. This can be easily accomplished with the popular analysis program PLINK [11]. It is harder to analyze multi-locus direct effects, where the specific alleles at a pair of loci modulates expression. It becomes computationally intractable to calculate indirect effects where a complex combination of an unknown number of alleles interact in affecting an expression level, or combination of expression levels. And of course, even if the raw statistics could be calculated, thousands or millions of ranked lists of millions of interacting SNPs and expression levels, with each list potentially depending on numerous factors, would be impossible to interpret directly.

Using the array of commonly available tools (summarized here [12]), only small slices of the eQTL visualization problem can be effectively tackled. The utility of such a piecewise approach is highly dependent upon the judgment and skill of the user, and the best way to approach this data and its analysis, is as yet undefined. Static or animated, fixed representation or interactive, exploratory or explanatory, displaying statistics, or guiding calculations to perform, it is hard to imagine any representation that cannot provide some useful insights into the data, and equally hard to imagine any that come close to being completely adequate for all uses. In the 2011 BioVis contest, entrants explored a large range of themes, and demonstrated tools that applied several of these themes.

### Judging

The specific question to be addressed by the contestants, was the elucidation and explanation of the factors, and the pattern of interaction amongst the factors, influencing the incidence of a particular phenotype. We conceived of this phenotype as a disease severity, for an invented disease, hoomphalitis. The incidence of hoomphalitis was influenced, but not strictly dictated, by the sum of the expression levels for the 8 genes in the spiked-in expression network. If the sum of the expression levels for these genes fell below a certain threshold, then that individual was 80% likely to be affected by hoomphalitis. If their summed expression levels exceeded the threshold, they were unambiguously unaffected. Contestants were specifically tasked with "Using the data provided, identify the pattern of genome-sequence variations, and expression-levels, that predict the occurrence of hoompalitis. To as great an extent as possible, elucidate and explain these factors, and the pattern of interaction amongst the factors, influencing the incidence of hoompalitis". A bonus question regarding a specific locus and a specific individual was also provided near the end of the contest. This question was "For a specific individual (person 1, family 425), if we were to modify his or her genotype at SNP rs12955865 to **TT**, what is your prediction regarding their affection status?". This question served as a test to see if the entrants could use the tools they had built, to answer a question that would be archetypical in the domain. Contestants were provided with eQTL data detailing 500 individuals, each genotyped at 7500 genomic loci, and with expression levels determined for 15 genes, as well as PLINK single-locus and two-locus analysis results for the entire dataset. The generation of this data is discussed in **Simulating eQTL data**.

Six judges (Team Vis: Tamara Munzner, University of British Columbia, Canada; Amitabh Varshney, University of Maryland - College Park, USA; Ananth Grama, Purdue Unversity, USA, and Team Bio: Mark Logue, Boston University School of Medicine - Biomedical Genetics, USA; R. Wolfgang Rumpf, Rescentris Inc., USA; and Shana Spindler, National Institute of Child Health and Human Development, USA) participated on two judging teams. Team Vis was asked to evaluate the entries based on whether they were using appropriate and innovative visualization/visual analytics approaches for analyzing and communicating the domain. Team Bio was asked to evaluate the entries based on whether they conveyed information that agreed with the experts' expectations and intuition regarding the biological patterns in the data. These tasks turned out to be considerably harder than anticipated. This was largely because our spiked-in data, incorporated into real biological eQTL data, provided knowledge of some effects that should be found, but not all effects that could be found, or knowledge of any effects that shouldn't be found. Furthermore, the goal of the contest combined both correctness and information transfer. The raw PLINK output could be considered to be completely correct, yet thousands of p-values in a file is undoubtedly inadequate for understanding the pattern of effects. Clearly, the judges needed to evaluate entries based on criteria beyond simple true and false positives and negatives.

Evaluating entries for this combined goal turned out to be one of the largest challenges for the judges. After considerable deliberation and discussion of how to evaluate specific features of entries, it was discovered that all members of Team Vis were in agreement on three entries that they felt displayed the most appropriate approach and innovation in the visual domain, and that all members of Team Bio were in agreement on three entries in which they felt the results agreed with biology, and for which they thought they could immediately use the presented tools in their research programs. Furthermore, there was a single entry that matched between these lists, and which both teams felt did an outstanding job in their respective domains. The entry selected by both judging panels was awarded the Overall Best Entry award, each teams top pick from their remaining favorites was awarded an Expert's Pick award, and the remaining selected entry from each panel awarded a special-mention award for the team's favorite characteristics in that entry.

### Simulating eQTL data

A major challenge in assessing the utility of novel analytical methods is posed by the trade off between having a known answer, which is created only by having a fully parameterized and specified simulated dataset that will lack many aspects of real biology, versus the natural complexity of real biological systems where the true depth and inner working remain at least partially hidden. Validation of analytical methods requires knowledge of what is in the dataset to assess sensitivity and specificity, making purely natural datasets less useful in this context, but a simulated dataset, however well-designed, may be too trivial to test the suitability of a method to for analyzing real data. The balance between these two competing virtues, specificity versus complexity, is therefore important to consider when designing a simulation to test methods, particularly when that data is being used for a contest.

We chose to simulate an eQTL network including three levels of complexity. First, genotypes and phenotypes were derived from two published eQTL datasets to ensure that natural relationships between the features were preserved. Second, a fully specified eQTL network was parameterized with a level of realism based on the experience of the data contributors to ensure that aspects of eQTL networks that scientific consensus indicates should exist, were present in the data. Third, model parameter values were chosen to be consistent with the observed datasets. The simulated data was "spiked-in" to data from the real eQTL datasets. This allowed the contest data to have several known features that could be extracted for comparisons and validation, but to also retain additional true biological relationships that were present in the data. Additionally, since biological data are inherently noisy, both from measurement error and the innumerable, apparently random fluctuations in biological systems, this contest design required entrants to identify the spiked in network in the context of real biologically generated noise found in the datasets underlying our simulation strategy. Our procedure, which is not typical of simulations in human genetics and was therefore implemented de novo here, represents a meaningful compromise between specificity and complexity.

### Real datasets

We used two datasets to obtain real eQTL relationships. The first dataset (Myers et al 2007 [13]) included 193 neurologically and psychiatrically normal postmortem human brain samples with a microarray assay that provides data on gene expression from all known genes and genomic data comprised of genotypes at 500,000 SNP loci. The second dataset (Liu et al 2010 [14]) consisted of 150 normal and psychiatrically diagnosed postmortem human brain samples with directly analogous gene expression and SNP data. For the contest, we used a subset of these data in the simulation. A total of 15 genes with gene expression and SNP data that passed standard quality control procedures [13,14] were selected from the cadherin protein superfamily, a class of proteins involved in cell-cell adhesion. Many of the 15 genes had previous evidence of interactions between them from other studies.

### Processing real datasets

For all subjects in the two studies, gene expression data from these 15 genes, as well as all SNP data within +/- 10,000 base pairs of each gene was used as the basis for simulation work. Since the gene expression data between the two datasets was not identically assayed (different microarray platforms were used) we applied a non-standard practice that we called "regularization" where data that was normalized within datasets as part of standard microarray gene expression data processing, is further standardized across datasets by subtracting the observed mean and then dividing by the observed standard deviation. The two datasets were then concatenated to create a "pooled" dataset. As both datasets were genotyped on the same platform, no additional processing steps were necessary except to exclude SNPs that did not pass quality control in each individual dataset. However, the number of SNP genotypes was far less than is representative of human genetic variation. Therefore we performed statistical imputation, where missing data are either inferred with certainty from the observed data or assigned upon the highest probability guess based on the observed data. In the case of SNP data, genotypes may be imputed based on the correlation between observed SNP data and SNPs in a reference dataset. SNPs retain correlation with other nearby SNPs on the same chromosome. Most normal human cells have two copies of each chromosome, and correlated SNP polymorphisms located on the same copy of a chromosome are said to be on the same haplotype. The imputation takes place when a correlated SNP haplotype in the observed data also correlates to a reference haplotype. If, as designed here, the reference haplotype has more SNPs than the observed data, the additional SNPs on the reference haplotype provide statistical guesses for those unobserved SNPs in the real dataset. We used reference haplotypes from the 1000 Genomes Project [15] dataset that included 61 persons with complete data (for our purposes) and an additional 38 persons with data only in coding portions of the genes (exome data) and none of the flanking sequence. The software MaCH was used for genotype imputation [16,17]. The final dataset was 7554 SNPs. As SNPs have only two possible values (called alleles) the frequency of which must sum to 1, we may characterize the informativeness of a SNP by reporting the allele frequency of one allele. By convention in genetics the smaller of the two frequencies, known as the minor allele, is reported. The average minor allele frequency over all SNPs was 0.17 with a total of 1557 having a minor allele frequency of 0, indicating that these polymorphism are so rare, they were not observed in our simulated dataset. The range of minor allele frequency was 0-0.5, thus the simulated dataset covers the full range of human variation in proportions observed in a real human dataset [15]. Lastly, as required for simulations below, several parameters were estimated. In each gene, a single SNP was chosen to influence gene expression for the spiked-in network. The average effect of each haplotype on that gene's expression was estimated by a series of linear regressions to obtain the partial effect of each haplotype, versus the average effect of haplotype substitution for that gene.

### Overview of the simulation

The simulation was conducted in two stages. The first stage was a data shuffling technique where two sets of haplotypes (one for each copy of a chromosome in human cells) across all genes were randomly assigned to a simulated person and a rejection procedure was implemented to ensure that the resultant gene expression data was consistent with the correlational structure of the observed data where the haplotypes were drawn. The second stage was preparation and integration of spiked-in data. Gene expression values for all 15 genes were simulated with a subset of gene participating in a gene expression network, parameterized in a 15 × 15 × 3 correlation matrix for all possible interaction of genes by pairs of alleles (also called genotypes).

### Data shuffling

As part of genotype imputation, the haplotypes of the observed data were estimated. Each subject's collection of haplotypes was stored along with observed gene expression values. A set of haplotypes, one for each gene, was randomly chosen with replacement from a randomly chosen subject, then a second set of haplotypes was independently chosen using the same procedure. Gene expression values consisted of the sum of partial expression values (above) for each haplotype selected for the simulated subject. A rejection procedure was implemented to ensure that the observed correlation in the simulated dataset was consistent with the observed correlation structure in the real dataset. As each set of gene expression values was simulated, it was added back to the real dataset and the observed correlation matrix was calculated. Deviations from the original observed correlation matrix of greater than 0.02 for any value was considered a rejected set of simulated values.

### Spiked-in network

**Figure 1:**
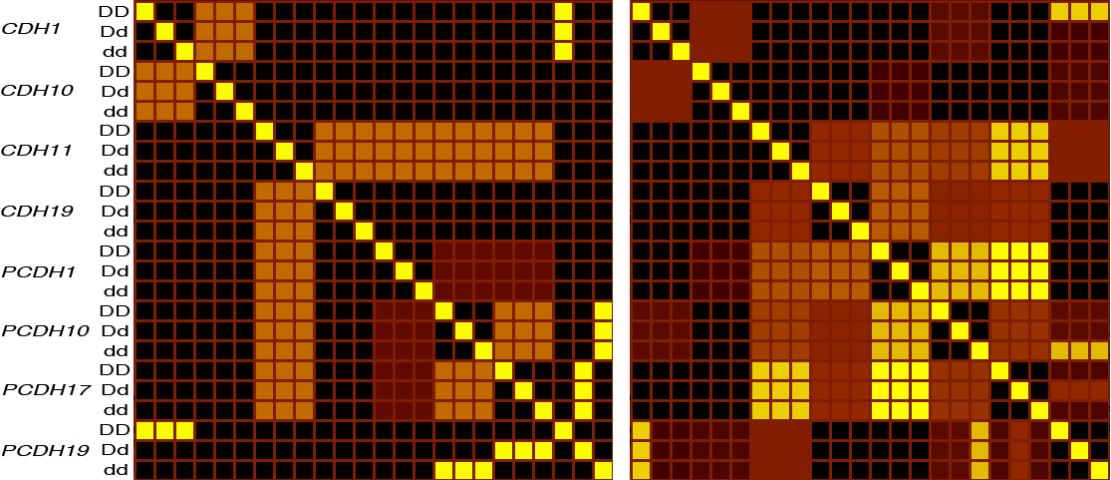
**A heat map representation of the spiked-in correlation network in the simulated data**. The heatmap is a two dimensional projection of a four dimensional matrix, 15 × 15 *genes *× 3 × 3 *genotypes*. Here the 3 × 3 cross-genotype blocks are nested within each gene block. As a self-correlation matrix, the column IDs are identical to the row IDs. The left panel shows the two sub-networks that were used to drive the simulation, one involving *CDH1 *and *CDH10*, the second involving *CDH19*, *PCDH1*, *PCDH10*, and *PCDH17*. *PCHD19 *interacted with several genes, but only under certain genotype configurations. This matrix also implies other high order dependencies that are not well shown in this form, but can be observed by tracing from a significant value in a cell, to any other significant value for another gene that occurs in either the same row or column. The number of steps along which such a chain may be followed, defines the number of interacting factors. The correlation matrix re-derived from the output of the simulation (right panel) includes both the spiked-in network and stochastic variation from the simulation, as well as the real biological correlations across genes.

The spiked-in network (Figure 1) was modeled as a series of correlations in a 15 × 15 matrix to express the *gene *× *gene *interaction, then an additional dimension was added in to allow for specific effects of the 3 possible genotypes at single SNP in each gene, where this single SNP was the only genetic variant in the gene that affects gene expression in the network (as described in Data processing section). The resulting correlation matrix, which due to our standardization procedures could be called a variance-covariance matrix, is not ideal for further statistical analysis since it not a properly formulated, symmetric positive definite matrix. Therefore the closest proper variance-covariance matrix was estimated [18] and used for the simulation. Using the R statistical language framework [19], the mvtnorm [20,21] library function "rmvnorm" was used to simulate random multivariate normal data using singular value decomposition on this variance-covariance matrix and genotypic means estimated in the data processing step (above). This simulation was conducted for each simulated person in the dataset conditional on the genotypes from the data shuffling step. The result is 15 gene expression values for each of 1000 simulated persons. The gene expression values were finally spiked-in by convolving the gene expression values from data shuffling with the spiked-in network multiplied by a weighting parameter. The weight of the spiked-in data was varied for each set of simulations where the spiked-in network was up-weighted in the first practice dataset (to make the network easy to find) and reduced on each consecutive iteration of practice datasets with the official contest data having the smallest value, and therefore these effects were harder to detect in the contest versus practice.

### Analysis of data available to all participants

We tested each gene expression-SNP pairs for association using standard linear regression (of allelic dose on expression) in PLINK [11]. We additionally ran every possible *SNP *× *SNP *× *phenotype *combination to assess *SNP *× *SNP *statistical interactions (non-linear effects) where the PLINK method incorporates an additional interaction term into the linear model and performs a test of significance on that term. All gene expression-SNP results were reported to contestants and all *SNP *× *SNP *× *gene expression *results with *p*-*value <*0.05 were also reported. Before data release, the weight of the spiked-in data was validated by ensuring that all of the *gene expression *× *SNPs *spiked-in were detected by PLINK analysis in the first practice dataset and that progressively fewer signals (but always more than zero) were detected in each data release with the contest data containing the fewest. Participants were encouraged to use their own analyses if they felt they could improve on the PLINK results.

### Additional data for bonus question

An optional contest feature involved the effect of a gene therapy change to an affected person's genotype and its effect on disease status, which models the promise of genomic medicine, yet to be realized. The question was "What happens to the affection status of Family 425, person 1 if we change their genotype for rs12955865 (in *CDH19*) to 'TT'? (Hint: Imagine that this is a gene therapy trial and we want to know the prospects of success.)" The disease status in the contest data was calculated by summing the gene expression values for 8 of the 15 genes, then applying a threshold for affection status, if the sum was below 2, the subject was defined as affected 80% of the time. In order to solve the bonus problem, we note that person 425-1 has a summed gene expression value of 1.97, just below the threshold for affection of 2.0. If we remove the effects of *CDH19 *by subtracting the partial effect of the original simulated *CDH19 *SNP, this person's summed gene expression value would be 2.8, which is above the affection threshold and therefore unaffected. Next we add back in the effect of a TT genotype for rs12955865 (in *CDH19*), which exerts effects as a normal distribution with mean = 0.957 and SD = 0.911. Integration of the normal probability density function yield a 29.69% chance that this person will remain affected and a 70.31% chance they will become unaffected.

### The entries

With 53 individuals signed up for the contest web forum and downloading the data, 9 teams containing 30 individuals submitted entries. Numerous approaches were demonstrated for analyzing the data and conveying the results, sometimes several per team, with some teams leaning more towards directly conveying specific interacting SNP and expression loci, and others leaning more towards conveying an overall picture of the interaction network, and enabling users to explore the network to discover specific interactions. The modalities ranged from one entry that did not apply any traditional visualization, and instead relied purely on textual presentation, to one entry that used a highly novel visualization method and visual analytics approach, which, despite confusing both the Vis and Bio judging teams with respect to its exact interpretation, was nonetheless quite favorably received. The final judges' evaluation of these entries displayed some interesting features, not the least of which that there was little correlation between the overall accuracy of the entries, or even potential accuracy, and their scoring. In no particular order, the following are the highlights of each entry and the judges' comments on them:

#### Zhou, Song, Wang and Zhu

This entry applied more sophisticated statistical techniques to the raw data, to identify deeper associations than were available from the provided PLINK analysis [22,23]. Using the regularization shrinkage method, this group applied multivariate multiple regression to reduce the dimensionality of the data to a subset of SNPs affecting expression, and to construct an association map between SNPs and genes [24,25]. Beginning with genetic correlation, they correctly identified the block-structure of the SNP-expression interactions, which they visualized as a Heat Map, and correctly deduced the stronger cis-acting nature of most in-gene SNPs on their gene expression levels. They applied hierarchical clustering to identify highly-correlated SNP groups associated with each gene, and Principle Components Analysis to isolate the most probable functional SNP within each cluster. Multivariate multiple regression was used to identify the specific effects of the selected SNPs on expression. The association maps were visualized as sparse network graphs. Their methods correctly identified the genes involved in the spiked-in correlation network, and many of the principal SNPs affecting these genes, as well as a few multi-SNP interactions. However, possibly due to isolation of only the first principle component, and possibly due to the cutoff applied to identifying clusters within their hierarchical clustering (*R*^2 ^= 0.9), their regression framework incorrectly predicted that the SNP modified in the Bonus question, was unassociated with any gene.

#### Younesy and Moller

This entry approached the gene expression-disease aspect of the problem independently from the SNP-gene expression aspect. Histogram-based approaches with single genes demonstrated that expression levels for both affected and unaffected individuals were approximately uniformly distributed. Two dimensional scatterplots of all combinations of genes however demonstrated that for some gene pairs, affected and unaffected populations could be partially separated. A linear model was therefore constructed using all 15 genes and 500 individuals, resulting in a solution for 16 fixed coefficients that predicted a bimodal distribution between affected and unaffected individuals. The 8 genes within the spiked-in correlation network were correctly identified in this model as those with the largest magnitude coefficients. An interface was developed to enable expert users to impress domain-knowledge on these coefficients, by deselection of known-non-involved genes, and recalculation of the implied coefficients. To identify SNP effects on gene expression levels, first-order interactions, and then second-order interactions between SNPs and expression levels were calculated. This process was conducted by discretization of each gene's expression into high, medium and low expression levels, and grouping of individuals based on this discretization. Consensus alleles were identified for the subpopulation in the high and low groups, and ratios calculated for each SNP for the probability of possessing the high group consensus in the high group to the probability of possessing the high group consensus in the low group, and of possessing the low group consensus in the low group to its prevalence in the high group. A log-log scatterplot of these ratios demonstrates that the majority of SNPs - these being ones which have the same probability of occurring in the high group as the low group - lie along a line. Significant outliers predict a deviation from equal probability. By plotting the magnitude of these outliers for each SNP, versus the gene-coordinates for each SNP, pictures of the relevant SNPs and their distribution across the genes were constructed. Second order interactions were specifically examined in the context of secondary silencing SNPs, which unfortunately were not a large feature of the spiked in data, resulting in the reinforcing secondary interactions present in the spiked signal being mostly overlooked. The method was sufficiently robust to enable a correct answer to the Bonus question, and was selected by the Team Vis for a special mention for ease of interpretation.

#### Sakai and Aerts

This entry provided two exploratory tools, one to investigate the effect of gene expression on the disease, and one to investigate the effect of SNP genotype on gene expression. The expression-disease tool provided an interactive interface using (modified) parallel coordinates [26], which presented all of the individuals and expression levels simultaneously, and enabled the user to identify relevant factors through a visual analytics paradigm. Simple differential histograms for each gene expression in affected and unaffected individuals, and coloring of each individual's trace based on affected or unaffected status, provided an interface to ordering the parallel coordinates. This approach enabled correct isolation of the spiked-in network, and its modulation of the affected status for individuals, by iterative re-ordering of the coordinates until the affected individuals and the differential properties of their expression levels were clustered within the display. The second tool provided an interactive display of the PLINK-predicted effect of each SNP on each RNA expression level, ordered by genomic locus, and superimposed with the difference in allele frequency between affected and unaffected individuals, as well as a Circos [27]/Mizbee [28] inspired circular display of two locus interactions. Although the entry identified relatively few of the spiked-in SNPs specifically, it did describe many features of the expression interactions that were associated with disease, and many combinations of SNPs that affected expression. It correctly identified the specific effect of the Bonus-question SNP on the gene containing it, but did not arrive at a correct conclusion regarding this gene's overall contribution to affected status. This entry was overwhelmingly selected by Team Bio as the entry that they would be most comfortable using immediately in their research work, and was selected for the Biology Experts Pick award for the contest. A more in-depth discussion of this entry, from Sakai and Aerts, follows in **Awarded Entries**.

#### Paquette and Lum

Using Ayasdi's data analysis and visualization tool, Iris, this entry employed a unique topology-discovery and exploration method to explore both SNP effects on gene expression levels, and gene expression levels on disease. Their method is based on visualization of the topology implied by the similarity of different subsets [29]. In the case of expression levels, the individuals were (multiply) clustered by gene expression, the clusters connected by edges when they shared an individual, and the resulting graph laid out in a force-directed manner. By coloring this graph differentially based on gene expression level, or by affected and unaffected status, significant predictors of differential membership were identified visually. Using the same paradigm, SNPs were laid out based on the similarity implied by pairwise mutual information, and colored by the mutual information between the SNP and the disease state, or by the F-statistic of ANOVA between the SNPs and each of the 8 genes identified as significant predictors in the gene-disease visualization. Interpretation of these visualizations involves the visual identification of "flares" within the displayed data, where the flares display generally consistent coloring internally, and differential coloration with respect to the remainder of the bulk data. This entry correctly identified the probabilistic effect of the SNP in the bonus question, and also suggested additional information and analyses that would be required to confirm the potential change in affection status. This entry was selected as the Overall Best Entry by the combined panel of Vis and Bio teams, and is presented in more detail by Paquette and Lum in **Awarded Entries**.

#### Jäger, Battke, Vehlow, Heinrich and Nieselt

This entry applied canonical graph-layout approaches (GraphViz [30]) to a filtered list of the provided PLINK one-locus and two-locus results, and iHAT [31], an in-house tool designed for visualizing Genome Wide Association Study (GWAS) data, to the SNPs that were common to both the single, and two-locus PLINK analyses. In iHAT, a heat-map type visualization was created using rows for each individual and columns for each SNP, with colors assigned according to the agreement between each SNP and the reference SNP in release 37.1 of the genome sequence. Additional columns were created for the metadata of affected status, and the gene expression levels for each individual. By sorting this display according to affected status, they correctly identified, though visual means, that no clear pattern of SNPs was differentially associated with affected versus unaffected status. The affected and unaffected groups were then aggregated, and the heat map reassigned with color based on the value (complete agreement, partial agreement, or complete disagreement with respect to the reference genome) most prevalent for that group in the column, and saturation based on the uncertainty of that consensus value. Visual filtering was then applied to identify the subset of SNPs that appeared differential between the groups. This filtering reduced that data to 29 SNPs of predicted relevance, and further, correctly identified the spiked-in subset of differential expression levels modulating affected and non-affected status. Interestingly, this group approached the answer to the Bonus question using different tools than they produced for their primary elucidation of the effectors of disease status. Starting with the 29 SNPs that they isolated as being the most highly predictive of disease status, they identified the subset of individuals with a similar profile to the bonus-question individual across these 29 SNPs, and the Bonus SNP, using their clustering tool Mayday [32]. This identified a single individual with an identical profile across these SNPs, who, like the individual indicated for the Bonus question, was affected. They then searched for individuals who matched the profile, including the proposed "gene therapy" change to the bonus SNP. This identified a different individual that matched the updated profile, who was unaffected. From this they correctly inferred the probable effect of the proposed change, from affected to unaffected. This entry was chosen by Team Vis for the Visualization Experts Pick award for the contest. A more in-depth discussion of this entry, from Jäger et al. is included in **Awarded Entries**.

#### Kreisberg, Lin, Erkkila, May, Bressler, Eakin, Rovira and Shmulevich

This entry applied Regulome Explorer[33] to the problem of elucidating multivariate nonlinear relationships within the contest data. The team applied a decision tree approach, supported by the RF-ACE[34] machine learning algorithm for discovering multivariate associations. Dimensional reduction was accomplished by growing an ensemble of decision trees, and rejecting features that did not participate in any tree. Random Forests were also used to identify features relevant to particular gene expression levels [35]. This approach correctly identified the 8 genes in the spiked-in interaction network, and furthermore correctly identified many of the spiked-in interactions between the expression levels, though it did not identify any of the cis-acting SNPs contained in these genes. It also identified a strong disease-related expression interaction that was not part of the spiked-in network. This interaction was not identified by any other team, but because the contest data was built with real biological variation, this finding cannot be considered a false positive, as it may be a natural feature of the underlying data to which this approach is more sensitive than those of the other entries. The primary visualization of the results was presented as a Circos [27]/Mizbee [28] type circular interaction diagram, with overlaid metadata. The RF-ACE machine-learning engine was unable to predict the likely change of affected status conveyed by the Bonus question SNP, though this may have been due to an overly stringent confidence threshold.

#### Keller

This entry took a self-proclaimed most-naïve approach to the analysis. Effectively, Keller considered the two locus results, which present pairs of SNP loci that affect some gene expression level, and the genes implicated by the single-locus results for each of the SNPs in the pair, as implying relationships between this set of genes. He visualized this data using simple force-directed graph layout methods. This approach produced a surprisingly accurate recapitulation of the subset of genes in the spiked-in interaction network, as it closely linked 7 of the 8 spiked in genes, and produced the sole stated observation of the underlying biological regulatory mechanism we were working with in the data - that of cadherin regulation of protocadherins. Keller then imputed directionality upon the edges based on a set of possible regulatory mechanisms that might exist if either one, or both of the genes in the single-locus results disagreed with the gene predicted in the two locus result. This directionality was used to re-position gene-nodes in pseudo-hierarchical form, emphasizing sources and sinks. Several additional "blobby" Hypergraph-based displays [36] were computed, showing genes as nodes, and variably imposing edges based on genes sharing SNPs in the single locus results, genes sharing gene-gene SNP pairs in the two locus results, and overlayed edges indicating both shared SNP results, and edges from the gene-concept lattice computed by Formal Concept Analysis [37]. Keller applied all of these tools in an iterative and exploratory manner, to identify patterns of apparent regulation in the data, and in fact met with surprising success in producing an actual biological interpretation. However, his results would not be conveniently replicated by another practitioner, due to the reliance on exploration and intuition in choosing the displays to construct and the concepts to analyze, and in fact he approached the submission as an exercise in testing the utility of the representations, rather than as a presentation of a proposed best approach. Nevertheless, Team Bio found his representational methods familiar in their similarity to a common representational idiom used in developmental biology training, and chose this entry for a special mention for clarity to the biologist based on similarity to familiar representations. Keller did not attempt to answer the Bonus question in his entry.

#### Fitzpatrick, Archambault, Shah and Shields

This entry demonstrated a considerable understanding of the underlying biology and biostatistical problems inherent in eQTL analysis, and applied sophisticated, traditionally domain-appropriate statistical methods to identification of cis and trans acting SNPs, including appropriate filtering of uninformative minor alleles, and multiple-testing correction. A linear regression model was used as a first-pass analysis to identify main effects. This was then extended to identify interacting eQTL effects. At the thresholds applied, this approach identified the main effects within the spiked-in expression network correctly, but did not capture the gene-gene, or SNP-SNP-gene interaction effects in this network, although they did correctly predict that there were no significant SNP-disease, or SNP-SNP-disease associations (the SNP effects on disease in our model being entirely driven by SNP modulation of expression in the context of other effects, rather than by SNP direct control of disease). The authors then applied the Tulip visualization framework [38] to visualize a node-link diagram consisting of both genes and SNPs as nodes, and SNP-gene, and gene-gene edges as implied by their regression. This diagram was then used in an exploratory fashion by filtering it based on subnetworks implied by particular genes. Per-gene scatterplots were also used, displaying differentially colored cis and trans SNPs, with each SNPs (X,Y) coordinates determined by the negative log of the SNP's association with disease, and the negative log of the SNP's association with the gene expression level. Taken together, the approach developed by this team enabled them to correctly identify both the genes present in the spiked-in expression network, many of the spiked-in SNPs, and to characterize the overall negative correlation between the spiked-in expression network and disease. This elucidation that downregulation of the spiked network predisposed individuals towards disease, was the sole specific and succinct statement of this paradigm observed by the judges. Despite this correct recognition, the effect of the SNP indicated in the Bonus question was accidentally characterized as decreasing the expression of a key gene, and therefore the bonus question was not answered correctly.

#### Chalkidis and Tremmel

This entry applied joint and conditional Mutual Information (MI) analyses [39], to measure the extent to which gene expression levels, and SNPs, were informative regarding disease affected status. The MI data was then used in developing communications channel models of the information transfer between SNPs and disease, and SNPs and expression levels. In constructing these models the authors point out an interesting observation; that the entropy of the data defines the maximum information that can be discovered about it, and that consequently, as information is gleaned, the amount of information remaining to be discovered can be quantified. The authors applied this idea to their channel models to determine the proportion of the knowable information being recovered with respect to the information transfer from gene expression to disease, based on different subsets of genes assumed to participate in the communication. By testing this for different subsets, they identified the subset of genes that provided the greatest fraction of information regarding disease, and the subset of SNPs that also provided the greatest information regarding each gene expression level, and regarding disease.

Interestingly, this was the only team to examine the question of whether the expression levels caused the disease, or whether the disease caused the expression levels. Applying a communications-theory derived data processing theorem [40], which states that the MI between state X, and a subsequent state Y in a Markov Chain, is at least as large as the MI between × and any state following Y, and the calculated MI between the SNPs and expression levels, SNPs and disease, and expression levels and disease, the authors correctly deduced that in our spiked-in model, SNPs drive expression, which subsequently affects disease.

The entry correctly answered the bonus question, and was awarded a special mention for correctly identifying the greatest number of actually known-positive main interaction effects amongst all of the entries. It however caused considerable consternation amongst both judging teams, as it presented the results entirely textually, and did not rely on Visualization for either analysis or presentation.

### Awarded entries

Three entries were selected by the Judging teams for awards as the Visualization Experts' pick, the Biology Experts' Pick, and the Overall Best Entry. The winning teams were invited to summarize their entries for this manuscript:

### Visualization experts' pick: Güter Jäger, Florian Battke, Corinna Vehlow, Julian Heinrich and Kay Nieselt

We present Reveal, a tool for visual analyses of eQTL data. The starting point of an analysis using Reveal is a list of SNPs and genes, and data from a patient cohort covering the presence of the sequence polymorphisms and the expression values of the genes, as well as PLINK results providing information on significant association between SNPs and SNP pairs and differences in expression. A graph is constructed such that each gene in the data set is represented by a node. For each gene the number of significant SNP pairs with one SNP associated with that gene is determined. Nodes of genes with at least one such pair are assigned a unique color, all other nodes are painted using a gray fill.

**Figure 2:**
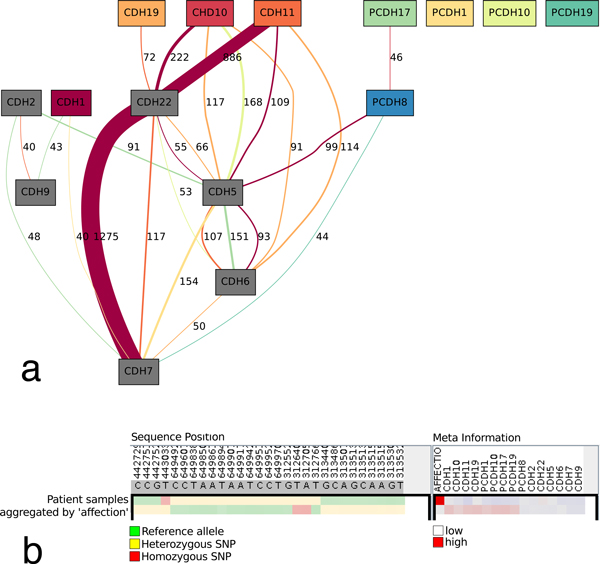
**The Visualization Experts' pick**. (a) Association gene network ed from all pairs of 3843 SNPs with a significant association (*p <*0.05, PLINK two-locus results) with the gene expression of the 15 genes and filtered such that only SNP pairs containing at least one highly significant SNP (*R*^2 ^*>*0.1 and *p <*0.05, PLINK single locus results) remain. All edges with weight *w *≥ 40 are shown. Nodes represent genes, edges represent significant SNP pairs. Genes significantly associated with SNP pairs are colored using a distinct color, genes with no significant association are drawn with gray fill. Each edge conveys four pieces of information: An edge *e *of weight *w *starting in node *s*, ending in node *t *and drawn with color *c *represents *w *SNP pairs, where each of them has one SNP in gene *s *and one in gene *t*. These SNP pairs are significantly associated with the expression of the gene whose node is filled with color *c*; (b) Aggregated iHAT visualization of 29 visually selected SNPs where the 'affected' and 'not affected' groups display different colors.

Edges are added between nodes as follows: Based on the *p*-values computed for the association between SNP pairs and gene expression, create a triple *< g_i_*, *g_j_*, *g_k _>*of genes for each SNP pair with partners in *g_i _*and *g_j _*that is significantly associated with the gene expression of *g_k_*. For each *g_k_*, add an edge between the nodes of *g_i _*and *g_j _*with weight *w *= |{*< g_i_*, *g_j_*, *g_k _>*}| and color *c*(*g_k_*). As SNPs located in, or close to, *g_i _*and *g_j _*can form pairs which influence the expression of different target genes, the graph can contain multi-edges which differ only in color, and possibly in weight. The resulting network is shown in Figure 2 (a). All SNPs represented in the network are then displayed in the association viewer iHAT [31] that supports the visualization of multiple sequence alignments, associated metadata, and hierarchical clusterings. Moreover, data-type dependent colormaps and aggregation strategies as well as different filtering options support the user in finding correlations between sequences and metadata. SNPs are colored green if both bases are identical to the reference sequence, yellow if one of the two alleles differs from the reference and red in the case that both alleles differ from the reference. Patient data included affection status (either affected 'red', or unaffected 'white'), is visualized as a meta information column. Furthermore, the gene expression data of the fifteen genes is also visualized as metadata using a color gradient blue-white-red representing low to high expression.

Next we sorted the column 'affection', resulting in the two groups of 'affected' and 'not affected' patients. Each group was then aggregated, with the aggregate value taken as the specific value observed with the largest relative frequency. The hue of the aggregated SNP value is chosen according to the color scheme for the SNPs described above, and the saturation and value of the color indicates the uncertainty of the aggregate consensus. By visual inspection we then filtered all those SNPs that displayed distinctly different colors between the 'affected' and the 'unaffected' groups (Figure 2 (b)).

### Biology experts' pick: Ryo Sakai and Jan Aerts

We present an exploratory tool for visual analytics in eQTL data. We performed minimal processing of the provided genotype and phenotype data and instead developed representations for the data in its original form. This decision was based on two factors: First, as the domain expert is already familiar with this type of data, he or she could interpret the visualization without learning new data-related concepts, and therefore could more readily interact and explore new hypotheses; Second, we believe that close interaction and iterative development in collaboration with domain experts is required for developing meaningful processing strategies, and the contest timeline could not accommodate this.

**Figure 3:**
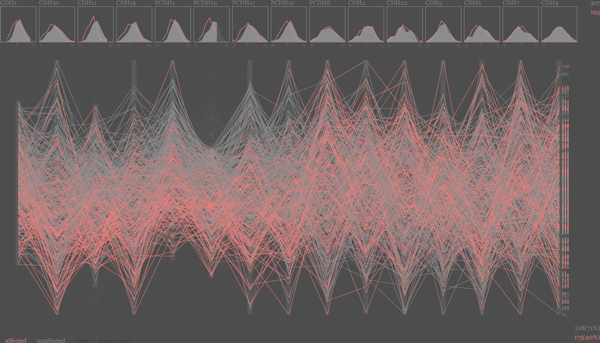
**The Biology Experts' pick**. Parallel coordinate display of gene expressions per individual. Vertical axes represent expression level for a given gene; horizontal polylines across the display represent each individual. Individuals are stratified in case (pink) versus control (grey). At the top of each vertical axis a histogram displays the distribution of expression levels of that gene over all individuals, stratified by group. The data for genes 1, 3, 5 and 6 are filtered for high and/or low values in this figure.

In order to explore and analyze the different aspects of the data, three different visualization modules were created. The first module (Figure 3) utilized parallel coordinates [41] defined by the fifteen gene expression levels to visualize each individual as a polyline. Different colors were used to distinguish cases from controls. A histogram was added for each axis/gene representing the distribution of gene expression levels, also stratified by case or control. Simple interactions and filter functions allowed the user to study combinations of different gene expression levels. These interactions included showing only cases or controls, filtering the expression values of any gene by value, and rearranging parallel coordinate axes. In addition, individuals could be filtered by any allele for any given SNP. The second module was targeted at exploring the single locus eQTL analysis data. The display consisted of a matrix of barplots; each bar representing the impact of a single SNP on a specific gene. This module clearly showed that the trans-interaction of single SNPs is limited in this dataset, although occasional signals were visible. The third module visualized the two locus data to study the networks of interacting SNPs that affect specific gene expression levels. Association between two SNPs is shown as lines within a circular representation, similar to that used in the Circos tool [27]. This representation clearly indicated groups of genes that are part of co-expression networks, including the known co-expression network of *CDH22 *and *CDH7*. Future work includes integrating these three modules into one cohesive visualization tool and conducting usability studies with domain experts to get insight to iterate both visual and interaction design for the analysis of eQTL data.

### Overall best entry: Jesse Paquette and Pek Lum

Our approach focused on visualizing the contest dataset with the Iris software platform (Ayasdi, Inc.), a topology-based exploratory analysis platform for complex datasets (http://www.ayasdi.com). Much as hierarchical clustering produces heatmaps and dendrograms showing how the points (rows) in a data set are related to each other over its dimensions (columns), Iris utilizes topology to capture geometric features in the data and presents relationships between points via interactive network maps. Topological methods often identify structures that elude linear clustering and projection [4,42,43]. Our primary goal was to produce a network map in Iris that visualized the effect of the SNPs on the expression of the 15 genes. From the contest-provided data, we produced a matrix *M *by calculating mutual information (MI) between all pairs of SNPs over all 500 patients. The matrix M was loaded into Ayasadi's Iris Platform [44] and a topological network map was constructed using the program's "Principal SVD lens" with resolution = 30 and gain = 3, and "Correlation Metric" [45].

**Figure 4:**
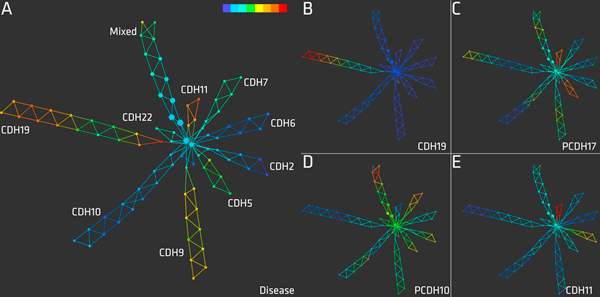
**The Overall Best entry**. A topological network map of SNPs produced by Iris. Each node represents a cluster of SNPs and nodes are connected with an edge if they have any SNPs in common. The starburst shape indicates subgroups of SNPs with distinct linkage disequilibrium patterns in the data set. A) Each flare of the starburst contains SNPs from a single locus and is labeled accordingly, except for the "Mixed" flare. The nodes are colored by SNP mutual information with disease. Higher mutual information values are colored red and indicate a stronger relationship. B) The nodes are colored by SNP ANOVA F-statistic with expression of *CDH19*. Higher F-statistics are colored red and indicate a stronger relationship. The flare with the red tip contains SNPs from the *CDH19 *locus; see label in A. C) The nodes are colored by F -statistic to expression of *PCDH17*. D) The nodes are colored by F -statistic to *PCDH10*. E) The nodes are colored by F -statistic to *CDH11*.

Figure 4 shows the resulting network maps of SNPs produced by Iris. Nodes in each map represent clusters of SNPs and edges indicate clusters that have at least one SNP in common. In other words, every SNP in the dataset can be located in more than one node. The size of each node is proportional to the number of SNPs it contains. Note the starburst shape in the SNP data, with large nodes at the middle and smaller nodes extending towards the tips of the flares. All of the flares in the starburst, except that labeled "Mixed", contain SNPs exclusively from a single locus and are labeled accordingly. For example, all of the SNPs in the *CDH10*-labeled flare are in the *CDH10 *locus. The single-locus flares recover an important pattern in the data: linkage disequilibrium (LD) between SNPs.

The exploratory power of Iris visualization comes from unsupervised construction of the network map, followed by coloring of the map using phenotype values; in this case the phenotypes for the SNPs are relationships with gene expression and disease. Figure 4 presents different colorings of the same network map; each color scheme shows how the SNPs relate to disease expression (Figure 4 panel A) or individual gene expression (Figure 4 panels B-E). The label in the bottom right of each panel indicates the color scheme source. The color of each node represents the mean of the statistic for all of the SNPs contained within. For the color scheme showing relationship to disease (Figure 4 panel A), a MI statistic was calculated for each SNP with respect to patient disease status. Larger MI statistics indicate more significant relationships; red nodes contain SNPs with the highest MI vs. disease. For example, in Figure 4 panel A, the flares labeled *CHD19 *and *CHD11 *have the highest relationship with disease. For each color scheme showing relationship to gene expression (Figure 4 panels B-E), an ANOVA F-statistic was calculated for each SNP with respect to each gene's expression. Larger F-statistics indicate more significant relationships; red nodes contain SNPs with the largest F-statistic vs. individual gene expression. In short, the flares with the warmest coloring are the most interesting. If the disease were simply a function of SNP profiles, then the starburst colored by disease relationships (Figure 4 panel A) would implicate SNPs in the *CDH11 *and *CDH19 *loci (the warm-colored flares) as important influencers of disease. However, given the assumption provided in the contest description that disease is a function of gene expression, and gene expression in turn is a function of SNP profiles, we turned our focus toward the relationships between SNPs and genes.

The network maps in Figure 4 panels B-E illustrate the relationships between SNP allelic patterns and gene expression. One can see genes with cis affecting SNPs (in Figure 4 panel B the red-colored flare with the highest F-statistic for *CDH19 *contains SNPs from the *CDH19 *locus), trans affecting SNPs (in Figure 4 panel C the red-colored flares with the highest F-statistic for *PCDH 17 *contains SNPs from the *CDH11 *and *CDH5 *loci), and very complex expression relationships (e.g. Figure 4 panel D). Insights gained from topological network maps with subsequent exploration of color schemes and flare structures can directly lead to hypotheses that can be taken back to the wet lab (or other datasets) and tested. For example, a researcher could identify distinct subsets of SNPs that relate to the expression of *PCDH17 *and then design assays to discover which of those were actually affecting *PCDH17 *expression, and which ones were simply in LD with them. Alternatively, transposing the *SNP *× *patient *matrix yields a network map of patients. We are extending our methods to other domains such as genome-wide association studies and functional-genomics data to uncover structure and yield new perspectives on these areas.

## Concluding remarks

*If the brain were so simple we could understand it, we would be so simple we couldn't *(Lyall Watson)

Judging the contest was only slightly less complex than the actual practice of science. While the spiked-in network provided some uniformity around which contestants answers could coalesce, there was not, nor was there intended to be, a simple all-or-none, well-defined solution. While some solutions were sensitive to the spiked-in networks, it is possible that ostensibly less sensitive methods are *more sensitive *to features in the already present eQTL network from the underlying biological data. We did, after all, preserve this real eQTL network in the data. Unfortunately, this single contest with its single dataset cannot fully characterize the properties of the approaches, just as any one experiment alone cannot rule out all competing theories. However, when additional control experiments are performed, it is possible to systematically rule out alternative explanations. In 2011 we applied the lesson that simulated data must be complex to be useful. In 2012 we will apply the lesson that multiple datasets analyzed in concert, to provide virtual control experiments and contrasts, can help us tease apart the spiked-in answers from the biological data in the background.

Additionally, the burgeoning size of datasets will be a challenge in the years ahead. For some visualization methods, more data implies greater power, and realistic problems will only become more exciting as more data becomes available. However, for others, the first realistic problem that will have to be overcome, with more data, will be the presence of more data. Today's eQTL experiment involves several megabytes per subject, and several thousand subjects. Tomorrow's eQTL experiment could easily involve several terabytes per subject. How small is too small to be realistic, and how large is too realistic to be useful? The BioVis community will soon have to decide.

## List of abbreviations used

IEEE: Institute of Electrical and Electronics Engineers; eQTL: expression Quantitative Trait Locus; SNP: Single Nucleotide Polymorphism; CFD: Computational Fluid Dynamics; GWAS: Genome Wide Association Study; MI: Mutual Information.

## Competing interests

JP and PYL are employed by Ayasdi Inc, the creators of the software used in their contest entry. CB, WR, LH, SYC, RS, JA, GJ, FB, CV, JH and KN declare that they have no competing interests.

## Authors' contributions

CB and WR designed, developed, and conducted the described contest, and prepared this manuscript. LH and SYC wrote and performed portions of the data simulation. RS and JA, JP and PYL, GJ and FB and CV and JH and KN wrote their respective sections of the Awarded Entries section.
